# Therapeutic Outcomes of Anti-VEGF Agents Versus Corticosteroids in Diabetic Macular Edema: A Comparative Review

**DOI:** 10.3390/ijms27031142

**Published:** 2026-01-23

**Authors:** Saranya Sanaka, Minzhong Yu

**Affiliations:** 1 College of Medicine, Northeast Ohio Medical University, 4209 St. Rt. 44, Rootstown, OH 44272, USA; 2 Department of Ophthalmology and Visual Sciences, University Hospitals Eye Institute, Case Western Reserve University, 11100 Euclid Ave, Cleveland, OH 44106, USA; 3 Cole Eye Institute, Cleveland Clinic Foundation, 2022 E 105th St., Cleveland, OH 44106, USA; 4 Department of Ophthalmology, Cleveland Clinic Lerner College of Medicine of Case Western Reserve University, 9501 Euclid Ave, Cleveland, OH 44195, USA

**Keywords:** diabetic macular edema (DME), anti-VEGF therapy, corticosteroids, central macular thickness, visual acuity

## Abstract

This structured narrative review compared the efficacy, durability, and safety of anti-vascular endothelial growth factor (anti-VEGF) agents and intravitreal corticosteroids for the treatment of diabetic macular edema (DME), with the aim of identifying patient- and disease-specific factors to guide individualize therapy. A comprehensive search of PubMed, Embase, the Cochrane Library, and ClinicalTrials.gov was conducted for studies published between January 2009 and November 2025, including randomized controlled trials, meta-analyses, and large observational cohorts with at least six months of follow-up. Visual acuity, anatomical outcomes, treatment burden, durability, and safety were extracted, and evidence quality was assessed using the GRADE framework. Eleven studies encompassing 1341 eyes were included. Anti-VEGF therapy consistently produced greater improvements in best-corrected visual acuity, particularly in treatment-naïve eyes and in patients with worse baseline vision, whereas corticosteroids achieved larger reductions in central macular thickness and significantly reduced injection burden because of longer durability. However, corticosteroid therapy was associated with higher rates of intraocular pressure elevation and cataract progression. In pseudophakic patients and in chronic or refractory DME, functional and anatomical outcomes were generally comparable between the two therapeutic classes. Combination therapy resulted in the greatest anatomical improvement but at the cost of increased ocular adverse events. Overall, anti-VEGF agents remain the preferred first-line treatment for most patients with DME owing to superior visual outcomes and a more favorable safety profile, while corticosteroids represent valuable alternatives in pseudophakic eyes, chronic or anti-VEGF–refractory DME, and cases with prominent inflammatory features, provided that careful monitoring for ocular adverse events is maintained.

## 1. Introduction

Diabetic macular edema (DME) remains a leading cause of visual impairment among working-age adults worldwide. Hyperglycemia drives a cascade of biochemical abnormalities—including activation of the polyol pathway, protein kinase C pathway, oxidative stress, and formation of advanced glycation end-products that disrupt the blood–retina barrier and promote extracellular fluid accumulation within the macula. VEGF-mediated vascular permeability and the upregulation of inflammatory mediators (e.g., IL-6, ICAM-1, MCP-1) contribute synergistically to retinal edema and progressive dysfunction of photoreceptors and the neurovascular unit.

Historically, focal/grid laser photocoagulation reduced the risk of moderate vision loss but rarely improved vision. The advent of intravitreal anti-VEGF therapy transformed the therapeutic landscape, offering significant vision gains for many patients. However, up to 40% of eyes demonstrate suboptimal responses to anti-VEGF monotherapy, revealing the multifactorial nature of DME and the importance of inflammatory mechanisms [1].

Intravitreal corticosteroids—triamcinolone, dexamethasone implants, and fluocinolone acetonide implants—target the inflammatory pathways central to chronic DME. These agents reduce cytokine expression, leukostasis, and VEGF production. Their extended durability can significantly lessen treatment burden. However, corticosteroids carry substantial risks, notably cataract progression and intraocular pressure elevation. Therefore, they remain second-line in most treatment algorithms but play a key role in specific clinical scenarios.

Given the distinct mechanisms, efficacy profiles, and risk profiles of these treatment classes, a comprehensive comparative synthesis is needed to support individualize therapy selection.

## 2. Methods

**Figure 1:**
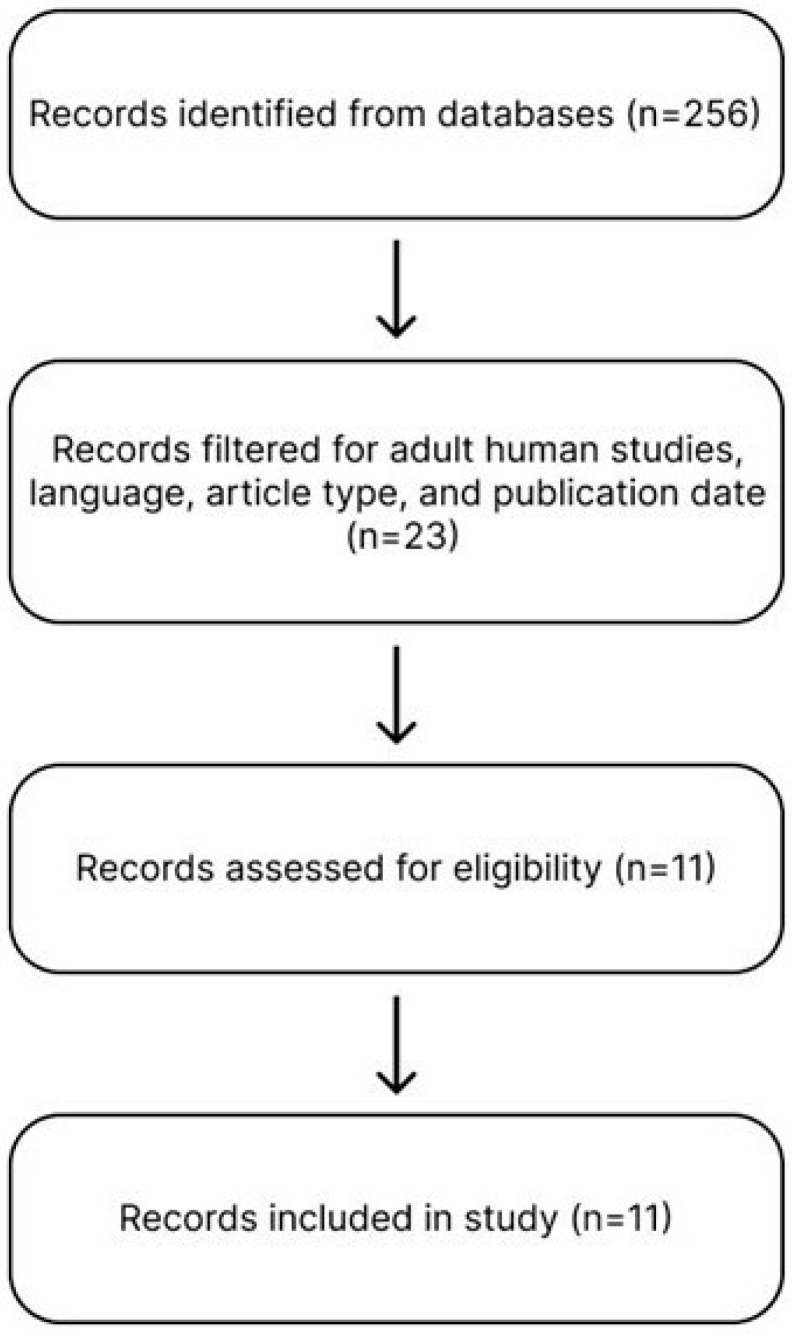
Flowchart illustrating the search strategy for studies included in this review. A total of 256 records were identified using the search string. After applying filters for adult human subjects, English language, publication within the past 15 years, and eligible article types (meta-analyses, observational studies, and randomized controlled trials), 23 studies remained for full-text assessment. Studies were excluded if they lacked a minimum follow-up of six months or did not report BCVA or OCT outcomes. Ultimately, 11 studies met all criteria and were included in the review.

A structured narrative review was performed through comprehensive searches of PubMed, Embase, the Cochrane Library, and ClinicalTrials.gov for publications from January 2009 to November 2025 (Figure 1). Systematic elements were included in the methodology to provide structure and improve transparency. A systematic review and meta-analysis were not conducted due to heterogeneity in study designs. Eligible studies included randomized controlled trials, meta-analyses, and large observational studies that evaluated anti-VEGF or corticosteroid therapies in adults with DME and had a minimum follow-up of six months. The search strategy used the following terms and their combinations: (“diabetic macular edema” OR “DME”) AND (“anti-VEGF” OR “bevacizumab” OR “ranibizumab” OR “aflibercept” OR “faricimab”) AND (“corticosteroid” OR “dexamethasone” OR “fluocinolone”) AND (“visual acuity” OR “OCT” OR “retinal thickness”). Bevacizumab (Avastin^®^; Genentech, South San Francisco, CA, USA), ranibizumab (Lucentis^®^; Genentech, South San Francisco, CA, USA), aflibercept (Eylea^®^; Regeneron Pharmaceuticals, Tarrytown, NY, USA), and faricimab (Vabysmo^®^; Genentech, South San Francisco, CA, USA) are anti-VEGF agents that are approved by the U.S. Food and Drug Administration (FDA) for ophthalmic use or widely used off-label in clinical practice, and are often used compared to other anti-VEGF agents. So, the search strategy focused on these agents.

After screening and qualitative assessment, eleven studies met the inclusion criteria and were incorporated into this review. Studies were limited to human subjects, English-language publications, and research published within the last 15 years. Data extracted from each study included author, publication year, sample size, study design, intervention arms, best-corrected visual acuity (BCVA) outcomes, optical coherence tomography (OCT) outcomes, safety data, and treatment frequency. The GRADE framework was applied to evaluate the overall quality and strength of the included evidence (Table 1).

## 3. Overview of Therapies

### 3.1. Anti-VEGF: Mechanism, Clinical Use, Limitations

Vascular endothelial growth factor A (VEGF-A) is a central mediator of retinal vascular hyperpermeability in DME. Anti-VEGF therapies, such as bevacizumab, ranibizumab, and aflibercept, bind circulating VEGF isoforms, preventing their interaction with VEGF receptors on retinal endothelial cells, primarily VEGFR-2. By inhibiting VEGF–VEGFR-2 signaling, these agents reduce endothelial fenestration and junctional complex disassembly, suppress downstream phosphorylation cascades including PI3K/Akt and MAPK pathways, and restore the integrity of the blood–retinal barrier. The clinical consequence is a rapid reduction in vascular leakage and retinal thickening, accompanied by secondary restoration of outer retinal architecture, which underlies many of the observed gains in visual acuity.

Newer agents such as faricimab further target angiopoietin-2 (Ang-2) in addition to VEGF, enhancing pericyte–endothelial interactions and mitigating pathological vascular remodeling. Bevacizumab, ranibizumab, aflibercept, and faricimab have received FDA approval or are widely used off-label for ophthalmic indications. Conbercept is another new anti-VEGF agent that has received the National Medical Products Administration (formerly the China Food and Drug Administration) approval and has received approval for Phase III Clinical Trials by the FDA.

VEGF itself is a secreted glycoprotein that plays a fundamental role in angiogenesis, the formation of new blood vessels from pre-existing vasculature [13]. While angiogenesis is critical during embryonic development and for normal wound healing in adults, VEGF levels are abnormally elevated in DME, contributing to dysregulated vascular permeability and abnormal retinal microvasculature [14].

### 3.2. Corticosteroids: Mechanism and Patient Selection

Corticosteroids, including triamcinolone, the dexamethasone implant, and the fluocinolone acetonide implant, exert their therapeutic effects by binding to intracellular glucocorticoid receptors and modulating gene transcription [15]. This interaction rapidly suppresses inflammatory signaling by inhibiting transcription factors such as NF-κB and AP-1, thereby reducing the production of proinflammatory cytokines and chemokines (e.g., IL-6, MCP-1, ICAM-1). Consequently, this decreases leukostasis, microglial activation, and Müller cell-mediated inflammation to help restore retinal homeostasis.

Corticosteroids also indirectly downregulate VEGF expression and promote re-establishment of tight junction proteins, including occludin and claudins, in both the retinal endothelium and the RPE, leading to improved stability of the blood–retinal barrier. These mechanisms collectively produce substantial reductions in macular edema, particularly when long-acting intravitreal implants are used, although their benefits must be balanced against known risks such as cataract formation and intraocular pressure elevation.

Inflammation plays a significant role in the development of DME. Inflammation in diabetic retinopathy is caused by damage-associated molecular patterns (DAMPs) which can affect and activate the immune system [16]. Microglia normally maintain homeostasis, but prolonged stimulation from DAMPs and microglia switching to an active phenotype result in an increased release of cytokines [16]. Müller cells are glial cells participating in retinal homeostasis that can become dysregulated in diabetic retinopathy and release cytokines and VEGF [17].

Elevated levels of inflammatory mediators, including ICAM-1, IL-6, and MCP-1, have been detected in the vitreous of patients with DME [18]. Upregulation of cytokines such as IL-6 contributes to intraocular inflammation and can further increase VEGF expression, exacerbating vascular leakage and edema [19].

Corticosteroids mitigate these inflammatory processes and also suppress VEGF production. In clinical practice, corticosteroids are delivered via intravitreal implants (e.g., dexamethasone and fluocinolone acetonide) or intravitreal injections (e.g., triamcinolone) [20].

Because of their known adverse effects, such as cataract formation or progression and elevation of intraocular pressure, corticosteroids are generally considered second-line therapy [21]. They are often reserved for patients who exhibit an inadequate response to anti-VEGF treatment [22].

Corticosteroids cause intraocular pressure elevation by inducing gene changes that alter the production and destruction of the extracellular matrix for the trabecular meshwork [23]. This increases outflow resistance and therefore intraocular pressure.

The pathophysiology of corticosteroid-induced cataract formation is not fully understood. One consideration is that corticosteroids may bind to proteins and cause conformational changes such as oxidation [24]. Other possible mechanisms include metabolic disturbances and accumulation of epithelial cells from effects not directly related to the lens [24].

Corticosteroids may offer particular benefit in pseudophakic patients with intraocular lenses. These individuals can experience heightened postoperative inflammation, especially after complicated cataract surgery, which may lead to pseudophakic cystoid macular edema and can further aggravate DME [25]. In such cases, corticosteroids may be a more suitable therapeutic option, as they address both inflammatory pathways and VEGF-mediated mechanisms.

## 4. Clinical Outcomes

### 4.1. Visual Acuity and Anatomic Response

Best-corrected visual acuity (BCVA) and central macular thickness (CMT) are the primary endpoints in clinical trials for DME. The goals of DME treatment are to enhance visual acuity and minimize macular swelling. BCVA is commonly measured in letters using standardized visual acuity charts, while anatomic response is assessed by changes in CMT from baseline to the end of therapy. CMT is most often quantified using optical coherence tomography (OCT), a non-invasive imaging technique widely used for diagnosing and monitoring retinal diseases [26].

Anti-VEGF agents can provide greater improvements in BCVA, largely due to their strong anti-permeability effects and their ability to restore outer retinal structure. By contrast, corticosteroids frequently result in greater CMT reduction owing to their direct modulation of inflammatory mechanisms underlying DME. Among the studies reviewed, four demonstrated superior BCVA outcomes with anti-VEGF therapy, while six reported no significant difference between treatment modalities, and none favored corticosteroids (Table 2). In terms of anatomic response, seven studies showed greater CMT reduction with corticosteroids or combination therapy, two reported no difference, and none favored anti-VEGF agents.

### 4.2. Duration of Effect and Injection Burden

Patient adherence is critical, as early and consistent treatment of DME is associated with better visual outcomes [27]. However, adherence can be influenced by anxiety and discomfort related to intravitreal injections. These injections may cause pain during administration and can lead to ocular irritation for up to 36 h afterward [28].

Anti-VEGF regimens require monthly or bi-monthly dosing, which challenges adherence. Corticosteroid implants provide sustained release over months (DEX: ~3–4 months; FAc: up to 36 months), reducing treatment burden substantially [2,4,5,8]. This advantage must be weighed against safety considerations.

## 5. Safety Profiles

The major safety concerns associated with corticosteroid therapy are elevations in intraocular pressure (IOP) and the development or progression of cataracts. These adverse effects are well recognized and often limit long-term use, especially in phakic patients. Systemic adverse events have been less thoroughly investigated, but current evidence suggests that their overall incidence is generally comparable between anti-VEGF agents and corticosteroids such as dexamethasone [29]. When systemic events occur, they most commonly involve cardiac or renal function and require appropriate clinical monitoring.

Regarding treatment outcomes, anti-VEGF agents typically provide greater improvements in visual acuity, while corticosteroids more often lead to larger reductions in central macular thickness. However, these anatomic benefits are accompanied by higher risks of ocular adverse effects. Corticosteroid-treated eyes showed significantly more cases of IOP elevation, with 304 affected eyes compared with 39 in anti-VEGF groups. Cataract progression was also more frequent in corticosteroid and combination therapy arms [8,9]. Overall, anti-VEGF therapy has a more favorable ocular safety profile, while systemic safety appears broadly similar between the two treatment classes.

## 6. Results and Discussion

### 6.1. Comparative Analysis and Interpretation

A total of 11 studies involving 1341 eyes were included in this review. Ten studies directly compared anti-VEGF therapy with corticosteroids or combination regimens, while one study evaluated corticosteroids in eyes that were refractory to anti-VEGF therapy. These studies included randomized controlled trials, prospective comparative studies, and retrospective cohort studies, with varying levels of certainty based on GRADE assessment (Table 1).

Across the included literature, anti-VEGF agents generally produced greater improvements in BCVA. Four studies demonstrated statistically significant BCVA advantages for anti-VEGF therapy, while six reported no significant difference between treatment classes. No study showed superiority of corticosteroids in improving vision. One noninferiority trial indicated that dexamethasone implants achieved BCVA outcomes within the predefined noninferiority margin relative to ranibizumab, although mean BCVA gains remained higher in the anti-VEGF arm. Taken together, these findings highlight the consistent functional benefit of anti-VEGF therapy, particularly in treatment-naïve eyes or in eyes with worse baseline visual acuity [2,6,9,11].

In contrast, reduction in central macular thickness favored corticosteroids [3,4,6,7,8,9,10,12]. Seven studies demonstrated significantly greater CMT improvement with corticosteroids or combination therapy, whereas two reported no significant difference and none favored anti-VEGF agents. An additional study found significant CMT reduction with corticosteroids without a comparison arm. These results reflect the strong anti-inflammatory effects of corticosteroids, which target inflammatory cytokines such as IL-6, MCP-1, and ICAM-1 in addition to partially suppressing VEGF expression. Their pronounced anatomic efficacy suggests that inflammatory activity plays an important role in many cases of chronic or refractory DME.

Treatment durability and burden differed markedly between therapies [2,5,8]. Anti-VEGF agents typically required monthly or bi-monthly dosing, contributing to challenges with adherence and variable real-world outcomes. Corticosteroid implants offered substantially longer duration of action, with dexamethasone implants lasting approximately three to four months and fluocinolone acetonide implants providing sustained release for up to three years. Reduced injection frequency is a clinically meaningful benefit for patients who struggle with frequent clinic visits or inconsistent follow-up.

Safety outcomes, however, favored anti-VEGF therapy. Across ten studies reporting IOP outcomes, 304 corticosteroid-treated eyes developed IOP elevation requiring medical therapy compared with 39 eyes treated with anti-VEGF agents. Cataract progression was also markedly more common in corticosteroid and combination therapy arms, particularly in phakic individuals. Systemic adverse events were infrequently and inconsistently reported but appeared similarly low across both treatment classes. Overall, corticosteroids carried a substantially higher risk of ocular adverse effects, limiting their use in certain populations [2,3,5,10,11].

Overall, the evidence indicates that anti-VEGF agents remain the preferred first-line option for most patients with DME due to their superior effect on visual acuity and more favorable ocular safety profile. Corticosteroids, however, represent an important therapeutic alternative in specific settings, including pseudophakic eyes, chronic or refractory DME, inflammation-predominant phenotypes, and in patients who cannot adhere to frequent anti-VEGF injections. Although corticosteroids offer stronger anatomic responses and reduced treatment burden, their use requires close monitoring for cataract development and IOP elevation. Personalized treatment selection—incorporating clinical characteristics, imaging biomarkers, disease chronicity, and patient-specific factors—remains essential for optimizing outcomes.

Across the studies, BCVA outcomes showed no statistically significant difference between treatment types in six studies. One additional study found no significant difference in BCVA, although corticosteroid therapy was not directly compared with anti-VEGF treatment. Three studies demonstrated a statistically significant advantage for anti-VEGF therapy, and one study reported a significant result (*p* < 0.001) supporting noninferiority of corticosteroids compared with anti-VEGF (Table 3).

For CMT outcomes, seven studies reported statistically significant reductions favoring corticosteroid or combination therapy. One study found significant CMT improvement with corticosteroids without a comparison to anti-VEGF. One study did not report CMT data, and two studies found no significant difference between treatment groups.

### 6.2. Safety Profile

Across all studies, 304 eyes treated with corticosteroids experienced intraocular pressure (IOP) elevation requiring medical management, compared with 39 eyes in the anti-VEGF group; most cases resolved with treatment.

In ten studies reporting IOP outcomes, the corticosteroid group consistently showed higher rates of IOP elevation. Five studies found this difference to be statistically significant, while the remaining five did not report statistical analysis. One study did not include IOP data. Corticosteroids cause increased intraocular pressure by altering the trabecular meshwork, which prevents outflow [23].

Maturi et al. noted that the most common adverse events in the combination dexamethasone DDS and bevacizumab arm were self-limited punctate keratitis and conjunctival hemorrhage [7].

Callanan et al. reported a statistically significant increase in the following ocular adverse events in the dexamethasone implant group: overall ocular adverse events, intraocular pressure elevation, cataract, subcapsular cataract, ocular hypertension, vitreous floaters, nuclear cataract, vitreous hemorrhage, and lenticular opacities [9]. The mechanism by which corticosteroids contribute to the formation of cataracts is poorly understood, but could be through binding to proteins and causing conformational changes [24].

The number of studies monitoring systemic adverse events was much fewer compared to the number of studies monitoring ocular adverse events. None of the studies were designed to identify systemic risk, but some studies still monitored systemic events.

The BEVORDEX study reported systemic adverse events but did not provide statistical comparisons between treatment groups. The most common systemic adverse event was worsening hypertension, occurring in 1 of 15 patients in the bevacizumab group, 2 of 19 in the dexamethasone (DEX) implant group, and 4 of 27 in the combination group. Chest pain occurred in one patient each in the bevacizumab and DEX implant groups. In the combination group, one patient experienced chest pain, one had a myocardial infarction, three developed congestive cardiac failure, and two had cerebrovascular accidents. The study also noted the potential for systemic adverse events associated with chronic VEGF inhibition, risks that may not be seen with corticosteroid therapy, referencing data from the RISE and RIDE trials [8].

### 6.3. Subgroup Analysis

Seven studies included pseudophakic patients. Other subgroups examined across the studies included patients with serous retinal detachment, hyperreflective foci, and those with an incomplete response to anti-VEGF therapy.

The FAME studies reported a statistically significant difference in BCVA outcomes between the two treatment groups overall; however, no statistically significant difference was observed in the pseudophakic subgroup or in eyes with chronic DME [11]. Inflammatory pathophysiology may play a more dominant role in patients with chronic DME, so this subgroup should be further studied to better individualize therapy. Imaging biomarkers, such as inflammatory cytokines, may be helpful in determining therapies.

Chakraborty et al. included only pseudophakic patients and reported that corticosteroid therapy produced similar improvements in both BCVA and CMT compared with anti-VEGF treatment [2]. The FAME studies likewise found no statistically significant difference in CMT outcomes among pseudophakic patients or in eyes with chronic DME [11].

Callanan et al. included 54 of 127 pseudophakic patients in the dexamethasone (DEX) implant arm and 62 of 120 in the ranibizumab arm [9]. In this subgroup, the DEX implant produced greater improvements in BCVA than ranibizumab, relative to the overall study population.

Ozsaygili and Duru evaluated patients with DME who had serous retinal detachment and hyperreflective foci [6]. Hyperreflective foci are found on OCT and can predict microglial activation, which is a contributor to inflammation in retinopathy (Shu). Ozsaygili and Duru found that BCVA outcomes favored anti-VEGF therapy, and CMT reduction favored corticosteroid therapy, which was similar to general conclusions across all the studies.

Combination therapy with corticosteroids and anti-VEGF agents consistently produced the greatest anatomic improvements but did not yield proportional BCVA gains and was associated with higher rates of IOP elevation and cataract formation [2,3,4,5,7,9,10,11,12]. This suggests that combination therapy may be most appropriate as a rescue strategy in selected refractory cases rather than as a routine first-line approach.

### 6.4. Real-World vs. Clinical Trial Data

Three studies were retrospective analyses of existing patient data, while the remaining studies were prospective randomized trials. Differences between real-world and clinical trial outcomes were evident. Retrospective real-world studies demonstrated greater variability in both BCVA and CMT outcomes, reflecting inconsistent treatment intervals, heterogeneous patient populations, and more advanced baseline disease. These disparities highlight the importance of considering both efficacy and the practical feasibility of treatment regimens when selecting therapy for individual patients.

### 6.5. Limitations

This review provides a narrative rather than a meta-analytic synthesis. The included studies demonstrated substantial heterogeneity in design, dosing regimens, and follow-up duration. Reporting of systemic adverse events was inconsistent, and few studies offered head-to-head comparisons involving newer anti-VEGF agents such as faricimab. Study designs also varied considerably: four incorporated combination therapy, one included only a single treatment arm, and one switched patients from a dexamethasone implant to intravitreal aflibercept, resulting in differing dosing frequencies across studies. No studies using faricimab met the inclusion criteria, highlighting the need for comparative trials evaluating this newer agent against corticosteroids. Adverse event monitoring and reporting were not standardized; several studies did not report systemic adverse events, and most did not provide statistical comparisons. For example, although some studies observed higher rates of cataract formation or progression in corticosteroid or combination groups, these differences were often not reported as statistically significant relative to anti-VEGF therapy.

Future directions may be to examine newer anti-VEGF agents and molecular biomarkers. Absence of newer anti-VEGF agents such as faricimab and conbercept in comparative studies is a knowledge gap in the comparison between corticosteroids and anti-VEGF agents. Molecular biomarkers may provide insight into the pathophysiology of individual patients’ disease, such as inflammatory markers, and encourage individualize therapy.

## 7. Conclusions

Corticosteroid treatments, including the dexamethasone implant, generally provide visual acuity and macular thickness outcomes comparable to those achieved with anti-VEGF therapy. Several studies reported greater reductions in macular thickness with corticosteroids, whereas others demonstrated superior BCVA gains with anti-VEGF agents. Although corticosteroids can reduce treatment burden through fewer injections and may be further optimized by emerging delivery approaches such as suprachoroidal administration, their use is limited by higher rates of cataract progression, intraocular pressure elevation, and other ocular adverse events. Anti-VEGF therapy, in contrast, maintains a more favorable ocular safety profile, although the long-term systemic implications of VEGF inhibition are not yet fully understood.

Corticosteroids play a particularly important role in patient subgroups where inflammation drives macular edema, such as pseudophakic eyes, refractory cases, or individuals with inflammation-predominant phenotypes. However, in other populations, the risks associated with corticosteroid therapy may outweigh their potential benefits.

Overall, both anti-VEGF agents and corticosteroids are effective treatments for diabetic macular edema. Anti-VEGF therapy remains the preferred first-line option because of its strong performance in improving visual acuity and its more favorable safety profile. Corticosteroids represent a valuable alternative for selected patients, offering superior anatomic responses and reduced injection burden in pseudophakic or refractory DME and in cases with inflammatory features. Their use, however, requires careful monitoring for cataract development and IOP elevation. Personalized treatment strategies that incorporate clinical phenotype, imaging biomarkers, and individual patient characteristics are essential. Future studies integrating molecular biomarkers may further advance precision therapy for DME.

## Figures and Tables

**Table 1:** Study types and certainty based on the GRADE framework.

Authors	Study Type	GRADE (Certainty)
Chakraborty et al. [2]	Retrospective comparative case series	Low
Gutiérrez-Benítez et al. [3]	Retrospective observational	Low
Barakat et al. [4]	Prospective, controlled, double-masked study	High
Hernandez-Bel et al. [5]	Retrospective observational	Low
Ozsaygili & Duru [6]	Prospective, randomized, controlled	High
Maturi et al. [7]	Prospective, single-masked, randomized, controlled	High
Gillies et al. [8]	Prospective, randomized, single-masked, clinical trial	High
Callanan et al. [9]	Prospective, randomized, parallel-group, noninferiority	High
Maturi et al. [10]	Prospective randomized, Phase 2 clinical trial	High
Singer et al. [11]	Two prospective, randomized, double-masked trials	High
Shah et al. [12]	Prospective, randomized, subject-masked	High

**Table 2:** Number of studies finding better improvement in each type of treatment.

Test	Statistically Significant Difference, Better Improvement in Anti-VEGF Group	Statistically Significant Difference, Better Improvement in Corticosteroid Group	No Statistically Significant Difference
BCVA (letters)	4	0	6
CMT (μm)	0	7	2

**Table 3:** Information on each of the studies included and discussed in this review.

Authors	Treatment Arm 1	Treatment Arm 2	BCVA	OCT	Safety
Chakraborty et al. [2]	Aflibercept	DEX or IVA Implant	Statistically significant (favoring anti-VEGF)	Not statistically significant	IOP AEs significantly higher in DEX group
Gutiérrez-Benítez et al. [3]	DEX implants after DME refractory to ranibizumab or combination therapy	N/A	Not statistically significant	Statistically significant	Statistical significance not reported, 21% had elevated IOP AEs treated with medication
Barakat et al. [4]	Aflibercept	Suprachoroidal CLS-TA and aflibercept	Not statistically significant	Statistically significant (favoring combination)	Statistical significance not reported, IOP and cataracts AEs higher in combination group
Hernandez-Bel et al. [5]	Aflibercept	DEX implant and aflibercept	Not statistically significant	Not statistically significant	Statistical significance not reported, IOP AEs higher in combination group
Ozsaygili & Duru [6]	Aflibercept	DEX implant	Statistically significant (favoring anti-VEGF)	Statistically significant (favoring corticosteroid)	Not provided
Maturi et al. [7]	Bevacizumab	DDS and bevacizumab	Not statistically significant	Statistically significant (favoring combination)	Statistical significance not reported, IOP AEs higher in combination group No serious systemic adverse events.
Gillies et al. [8]	Bevacizumab	Dexamethasone implant	Not statistically significant	Statistically significant (favoring corticosteroid)	Statistical significance not reported. IOP adverse events greater in corticosteroid group Cataracts greater in corticosteroid group Systemic adverse events reported.
Callanan et al. [9]	Ranibizumab	DEX Implant	Statistically significant (favoring anti-VEGF). From confidence interval, DEX implant was noninferior.	Statistically significant (favoring corticosteroid)	IOP AEs significantly higher in corticosteroid group. Cataract AEs significantly higher in corticosteroid group.
Maturi et al. [10]	Ranibizumab	Dexamethasone and ranibizumab	Not statistically significant	Statistically significant (favoring combination group)	IOP AEs significantly higher in combination group. Cataract AEs did not have statistically significant difference. No serious systemic adverse events.
Singer et al. [11]	Ranibizumab plus deferred laser	FAc	Statistically significant (favoring anti-VEGF) Not statistically significant in pseudophakic and chronic DME eyes	Not provided	Statistical significance not reported, IOP AEs more in corticosteroid group
Shah et al. [12]	Bevacizumab	Dexamethasone delayed delivery system	Not statistically significant	Statistically significant (favoring corticosteroid group)	IOP AEs significantly higher in corticosteroid group. Cataract AEs did not have statistically significant difference. Systemic adverse events did not have a statistically significant difference.

## Data Availability

Data is contained within the article.

## Abbreviations

Ang-2	angiopoietin 2
Anti-VEGF	anti-vascular endothelial growth factor
BCVA	best-corrected visual acuity
DAMPs	damage-associated molecular patterns
DME	diabetic macular edema
IOP	intraocular pressure
OCT	optical coherence tomography
VEGF	vascular endothelial growth factor
VEGF-A	vascular endothelial growth factor-A
